# Supplementary material for: Age differences in electrocortical dynamics during uneven terrain walking
Source: Imaging Neurosci (Camb). 2025 Nov 26;3:IMAG.a.1039. doi: 10.1162/IMAG.a.1039 (PMC12658775; doi:10.1162/IMAG.a.1039)
Supplement: Supplementary Material [file IMAG.a.1039_supp.pdf]

Supplementary Material

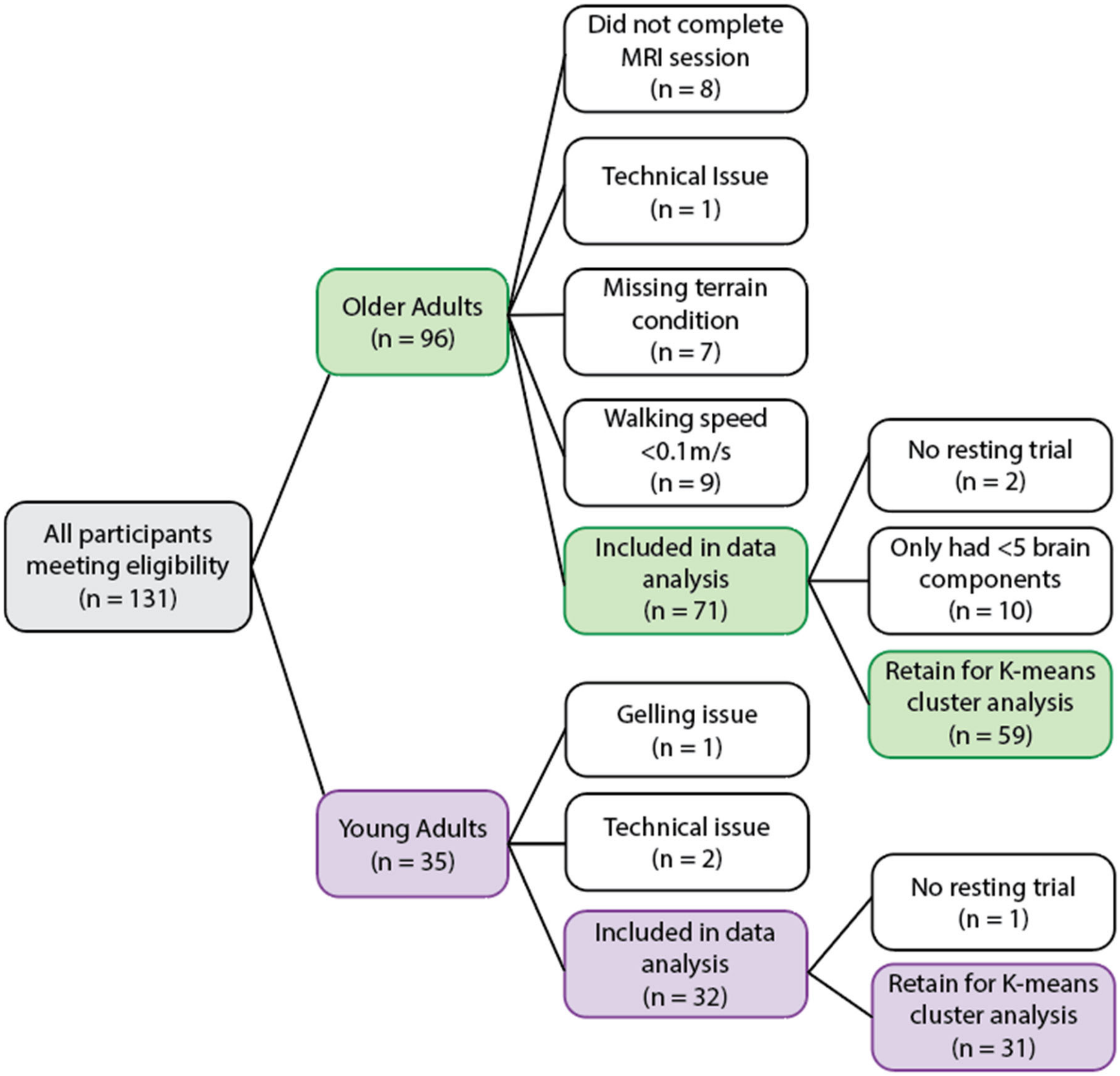

Supplementary Figure 1: Flow diagram of participant inclusion. We recruited a total of 35 young adults and 96 older adults for the EEG assessment. Among them, a total of 3 young adults were excluded for data analysis due to gelling issues with braids (n = 1) and 2) technical issues (n = 2). We further excluded 12 older adults and 1 young adult during data analysis. 25 older adults were excluded from data analysis due to 1) not completing the MRI session (n = 8), 2) a technical issue occurred during data collection (n = 1), 3) not completing high terrain conditions (n = 7), or 4) walking with a speed slower than 0.1m/s because these participants tended to walk toward the front of the treadmill, paused their walking, and then let the treadmill transport them toward the back.

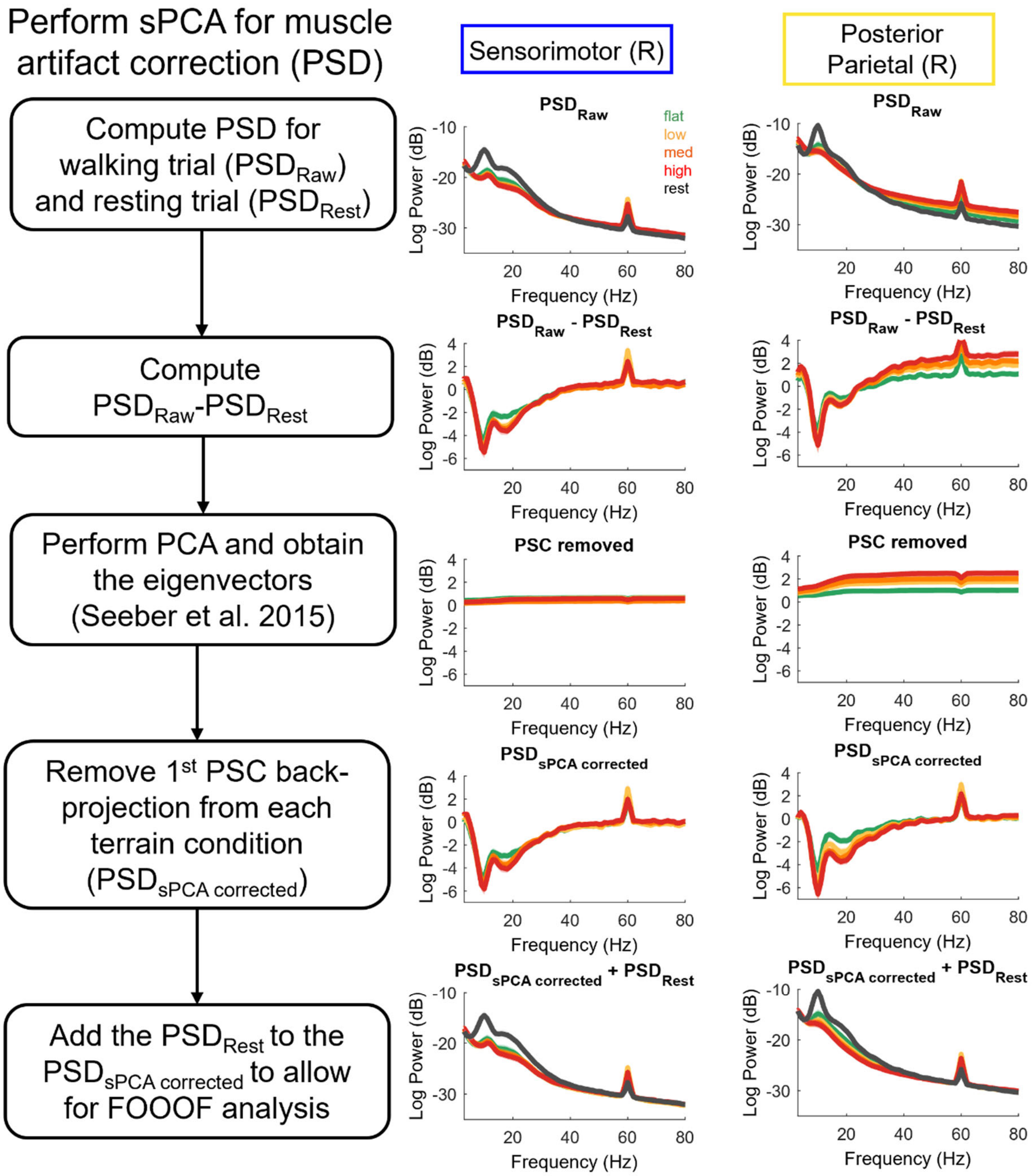

Supplementary Figure 2: Flow chart of spectral Principal Component Analysis for muscle artifact correction for the power spectral density analysis, along with the corresponding power spectral density plots at each step for the sensorimotor and posterior parietal areas. After obtaining the sPCA-corrected PSD, we added the PSD from the resting condition to the sPCA-corrected PSD to allow the FOOOF toolbox to separate the aperiodic and periodic components. PSC: principal component.

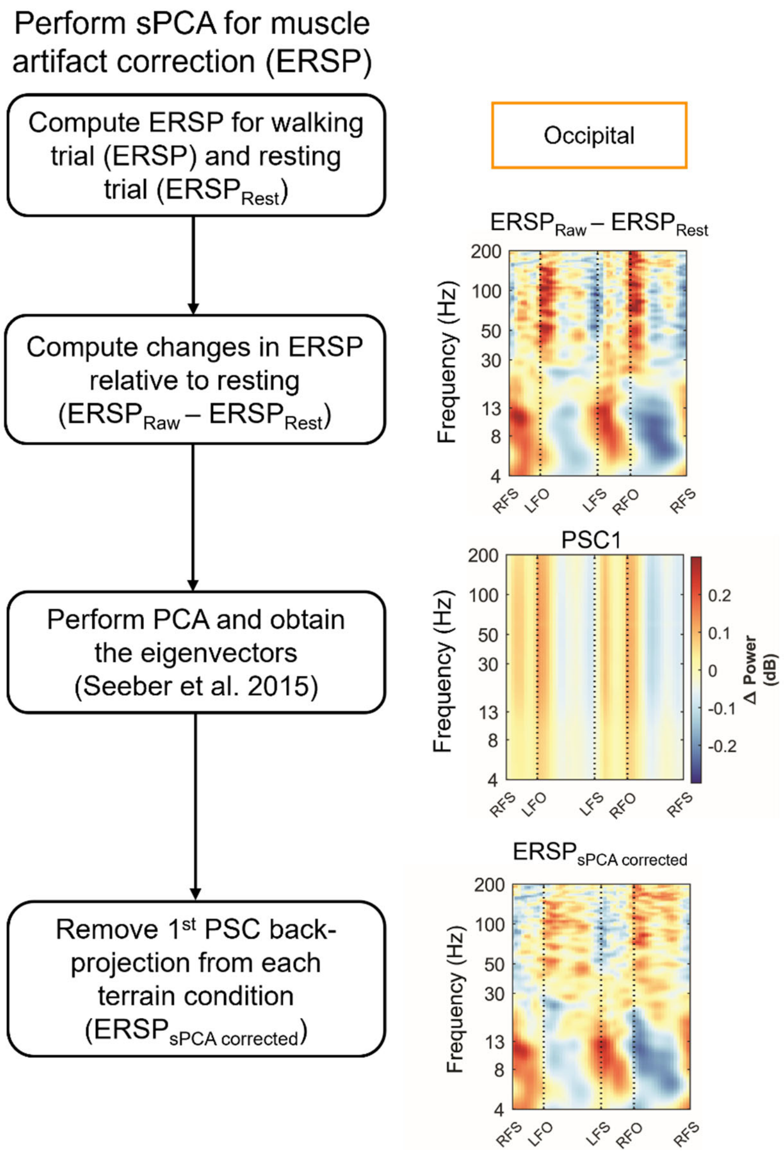

Supplementary Figure 3: Flow chart of spectral Principal Component Analysis for muscle artifact correction for the event-related spectral perturbation (ERSP), along with the corresponding ERSP plots at each step for the occipital cluster where the most muscle activity contamination occurred. PSC: principal component.

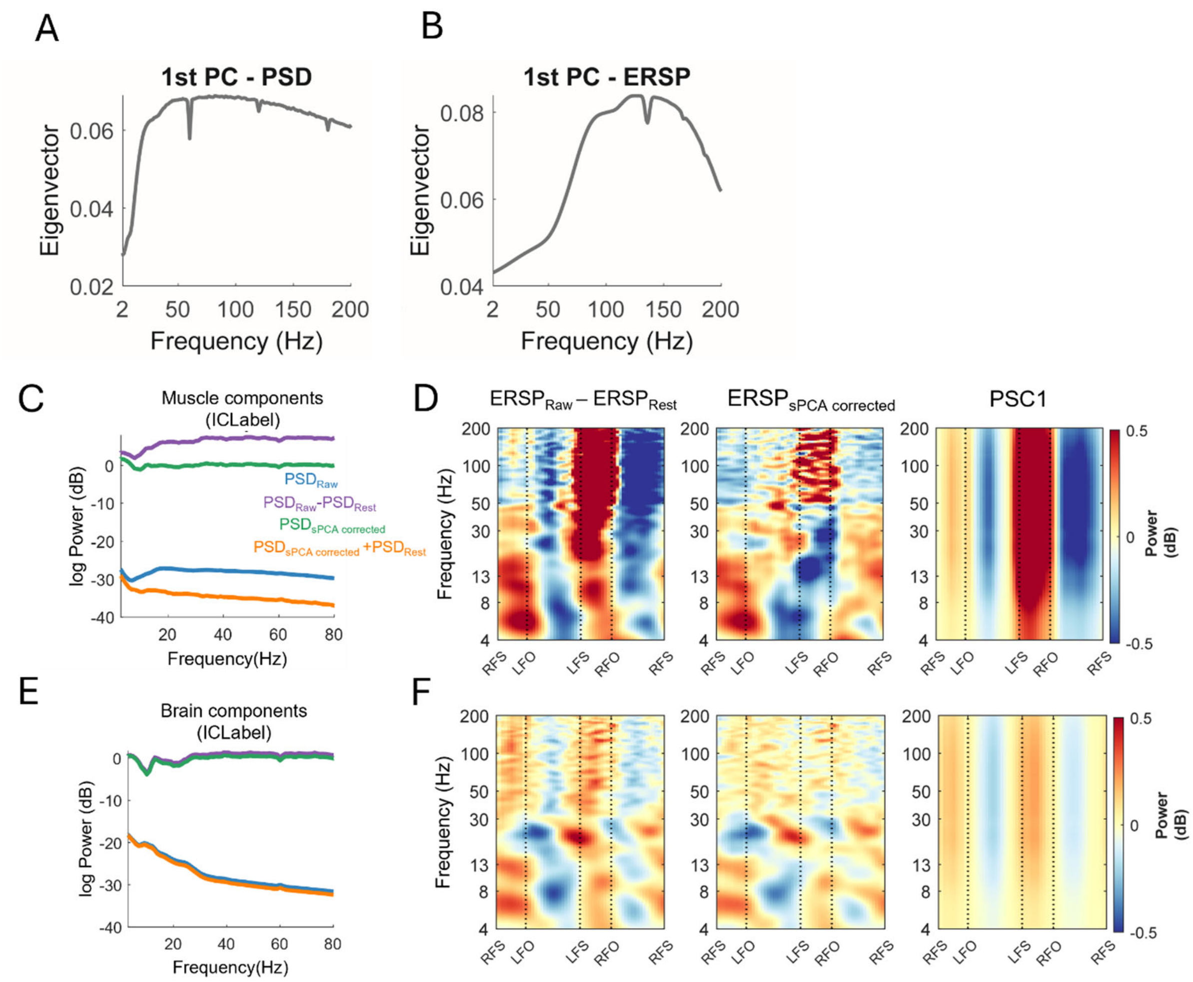

Supplementary Figure 4: Characteristics of the first spectral Principal Component. (A) First eigenvector for the power spectral densities (PSD; subject mean). (B) First eigenvector for the event-related spectral perturbations (ERSP; subject mean). (C) Averaged uncorrected and corrected PSDs for muscle components identified with ICLabel for one example participant. (D) Averaged uncorrected ERSP, sPCA corrected ERSP, and removed PSC1 for muscle components identified with ICLabel for one example participant. (E) Uncorrected and corrected PSDs for brain components identified with ICLabel for one example participant. (F) Uncorrected ERSP, sPCA corrected ERSP, and removed PSC1 for all brain components identified with ICLabel for one example participant.

Because the muscle components were corrected far more than the brain components, and the PSC1s mostly contained higher frequencies, we can reasonably conclude that PSC1 is primarily an electromuscular artifact.

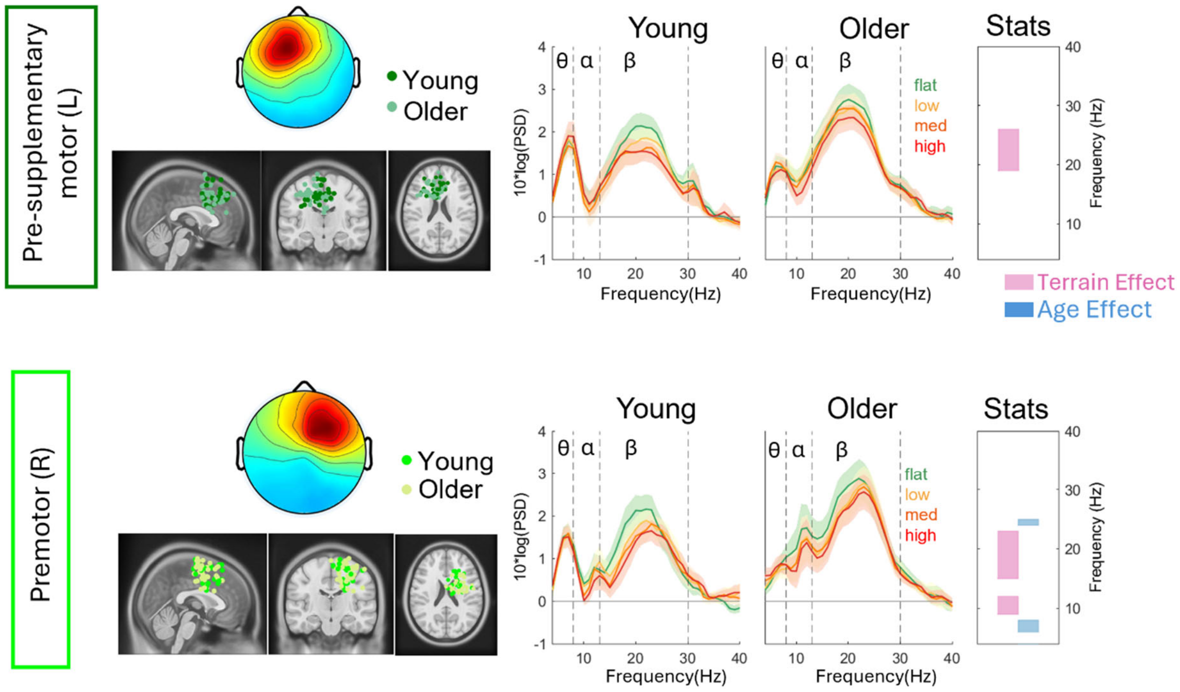

Supplementary Figure 5: Dipole location plotted on the Montreal Neurological Institute template, scalp topography, and average flattened power spectrum densities (PSDs) changed with terrain unevenness for younger and older adults for the pre-supplementary motor and premotor area. Shaded colored areas indicate standard error of PSDs across components in the cluster. Vertical black dashed lines indicate main frequency bands of interest—theta (4 - 8 Hz), alpha (8 - 13 Hz), and beta (13 - 30 Hz). The very right panel indicates the significant terrain and age effect on PSD at each frequency (pink: terrain, blue: age). In this non-parametric statistical analysis, only main effects were tested due to the limitation of this test.

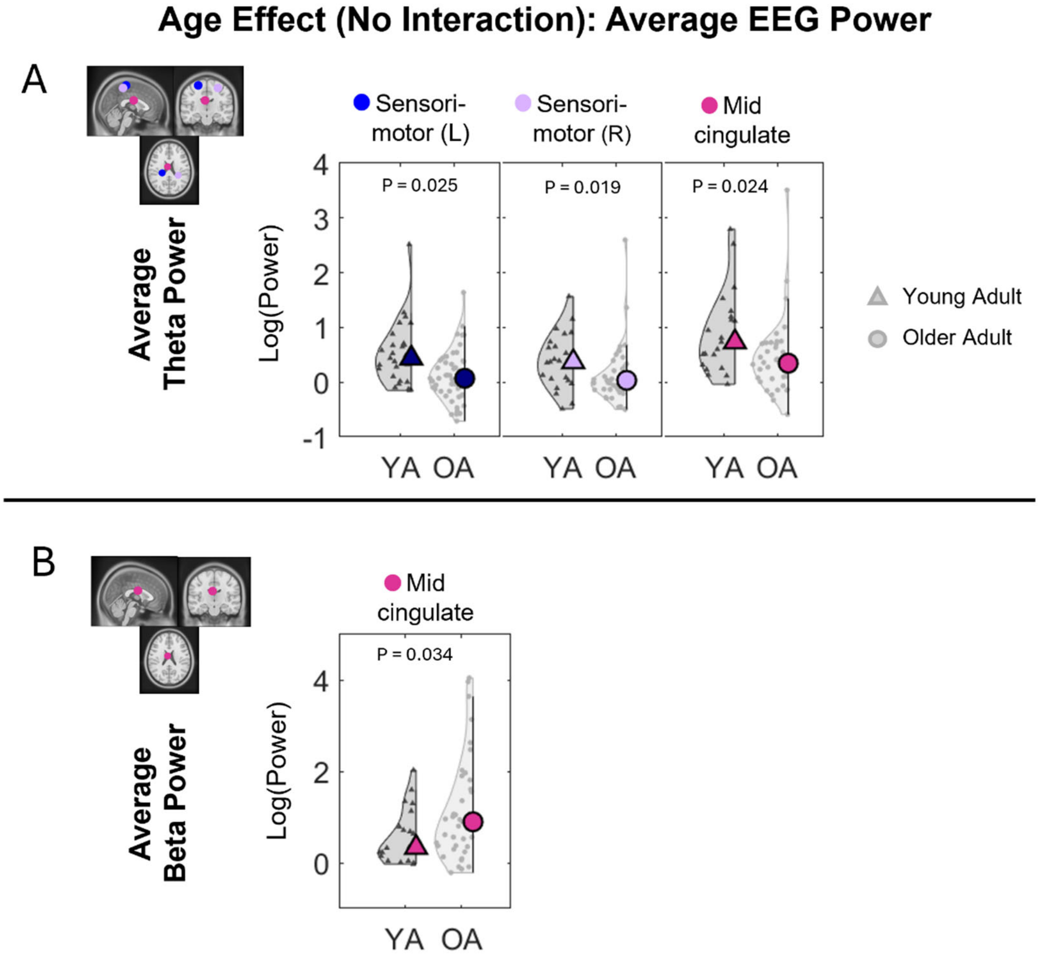

Supplementary Figure 6: Violin plots of the average alpha power (A) and beta power (B) for each age group after pooling all the terrain data for the corresponding brain clusters without an interaction effect. YA: younger adults, OA: older adults. Circle and triangle markers indicate median across participants.

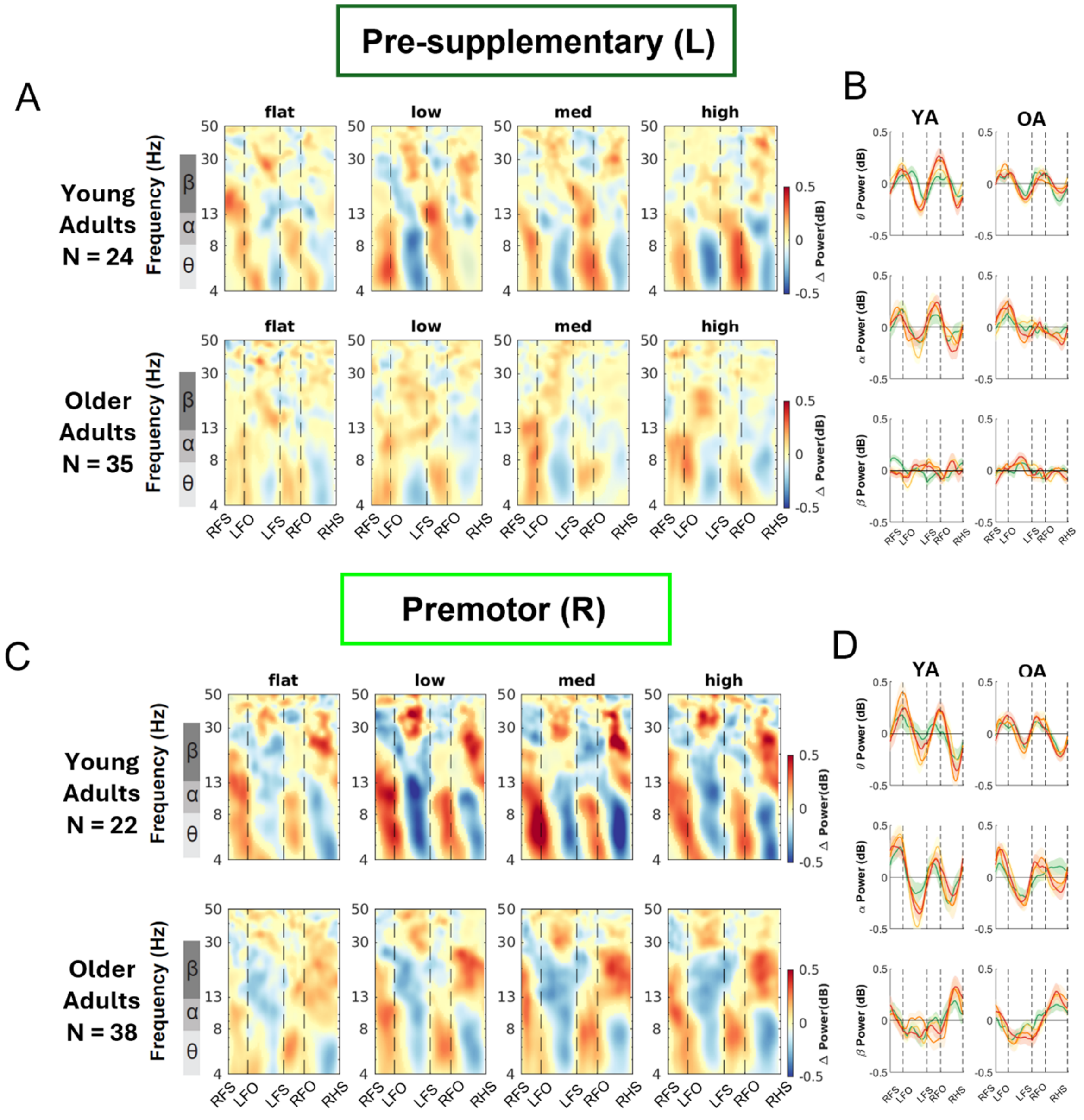

Supplementary Figure 7: Intra-stride event-related spectral perturbations (ERSPs) with respect to the average of condition at different terrains for young and older adults at (A) pre-supplementary and right premotor area (B), average intra-stride power fluctuations for each band at the (C) left pre-supplementary and right premotor area (D). (A, C) The ERSPs were displayed with the gait cycle on the x-axis (RFS: right foot strike; LTO: left toe off; LFO: left foot off; RFO: right foot off). All unmasked colors are statistically significant spectral power fluctuations relative to the mean power within the same condition. Colors indicate significant increases (red, synchronization) and decreases (blue, desynchronization) in spectral power from the average spectrum for all gait cycles to visualize intra-stride changes in the spectrograms. These data are significance masked ($p < 0.05$) through nonparametric bootstrapping with multiple comparison corrections using false discovery rate. (B, D) Average spectral perturbations for each band (theta, alpha, beta) across the gait cycle for young and older adults. YA: young adults. OA: older adults.

Age Effect (No Interaction): Intra-stride Power Fluctuations

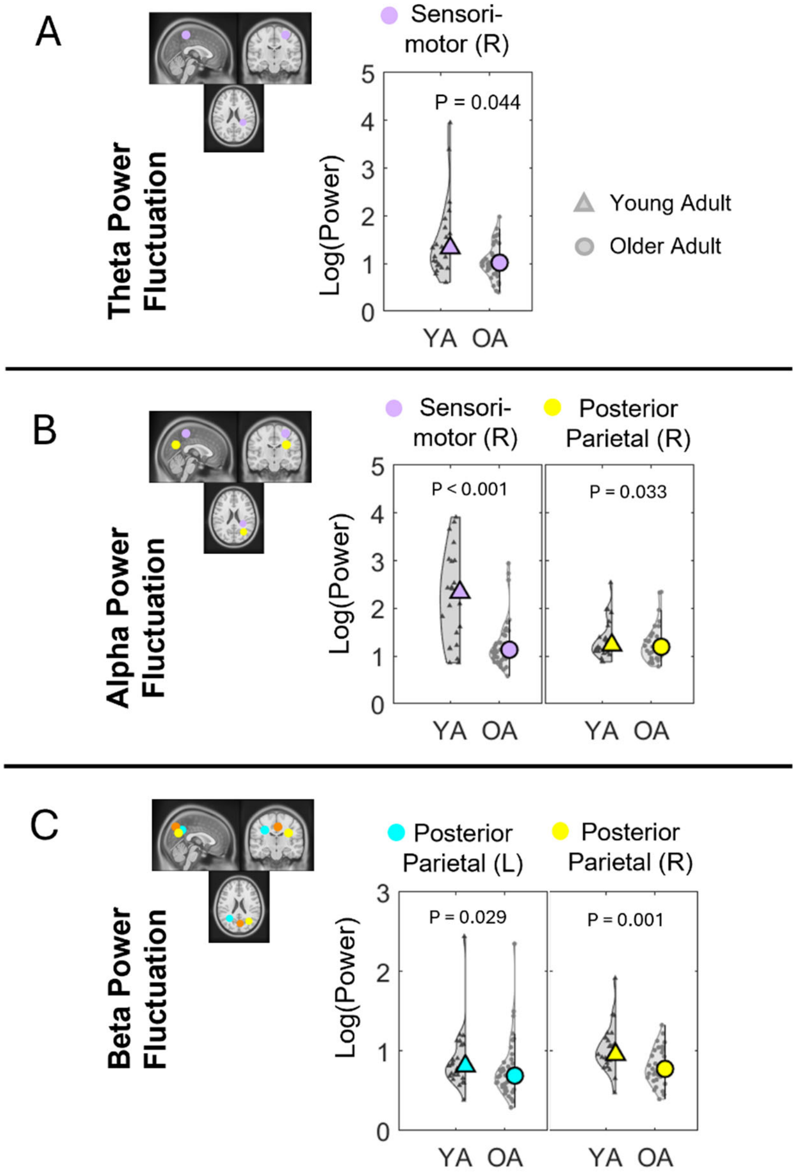

Supplementary Figure 8: Violin plots of the theta power fluctuations (A), alpha power fluctuations (B), and beta power fluctuations (C) for each age group after pooling all the terrain data for the corresponding brain clusters without an interaction effect. YA: younger adults, OA: older adults.

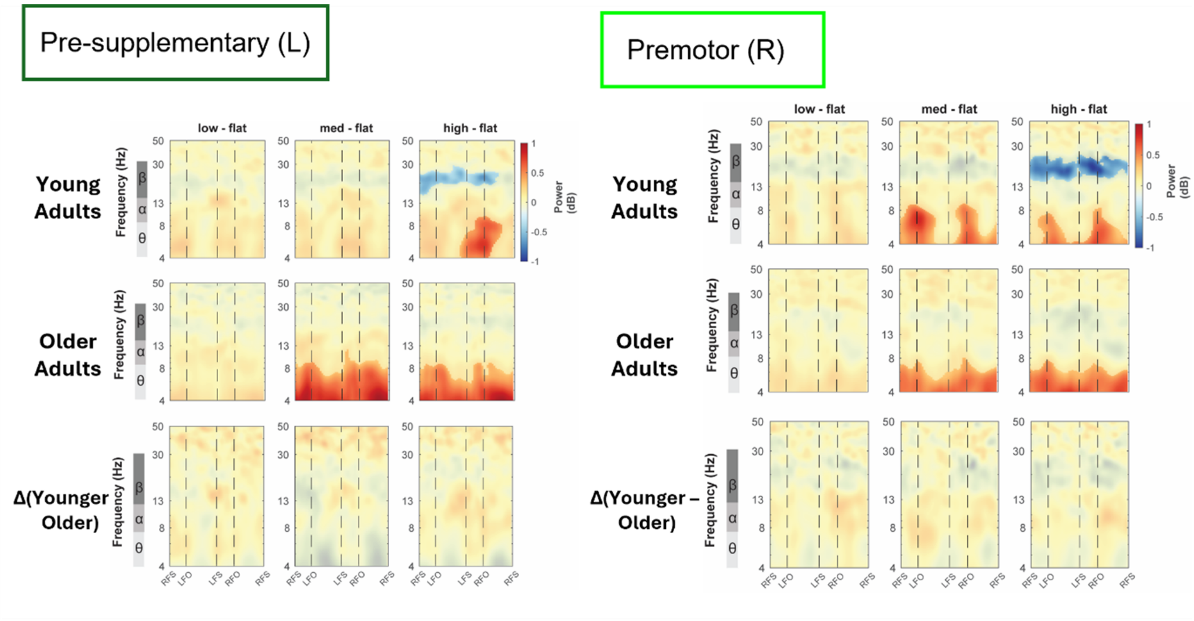

Supplementary Figure 9: Average event-related spectral perturbations (ERSP) with respect to the flat condition for each group and the difference between groups in the left pre-supplementary and right premotor. All unmasked colors are statistically significant spectral power fluctuations relative to the mean power within the same condition. The x-axes of the ERSPs are time in the gait cycle (RFS: right foot strike; LFO: left foot off; LFS: left foot strike; RFO: right foot off).

Supplementary Table 1: Statistical table examining the effect of terrain, age, and the interaction on each behavioral outcome (*p<0.05, **p<0.01, ***p<0.001).

Measure	Effect	Fstats	dF	dF(res)	Pvalue	Sig	Partial eta^2
Step Duration CoV	Terrain	28.33	3	297	<0.001	***	0.22
	Age	0.01	1	166	0.920		0
	Treadmill Speed	66.84	1	98	<0.001	***	0.41
	Terrain:Age	5.19	3	297	0.002	**	0.05
Anteroposterior Excursion CoV	Terrain	88.14	3	300	<0.001	***	0.47
	Age	43.81	1	98	<0.001	***	0.31
	Treadmill Speed	25.54	1	98	<0.001	***	0.21
Mediolateral Excursion CoV	Terrain	10.47	3	297	<0.001	***	0.1
	Age	0.30	1	145.09	0.585		0.002
	Treadmill Speed	10.80	1	98	0.001	**	0.1
	Terrain:Age	3.99	3	297	0.008	**	0.4

Supplementary Table 2: Multiple comparison results for step duration CoV with significant interaction effect (*p<0.05, **p<0.01, ***p<0.001).

Measure	Group	Terrain	dF	t	pFDR- adjusted	Sig
Step Duration CoV	YA	Flat v. Low	297	-4.21	<0.001	***
		Flat v. Med	297	-6.44	<0.001	***
		Flat v. High	297	-8.85	<0.001	***
		Low v. Med	297	-2.23	0.027	*
		Low v. High	297	-4.64	<0.001	***
		Med v. High	297	-2.41	0.027	*
	OA	Flat v. Low	297	-6.87	<0.001	***
		Flat v. Med	297	-11.16	<0.001	***
		Flat v. High	297	-19.66	<0.001	***
		Low v. Med	297	-4.29	<0.001	***
		Low v. High	297	-12.79	<0.001	***
		Med v. High	297	-8.50	<0.001	***

Band	Terrain	Group	dF	t	pFDR- adjusted	Sig
Step Duration CoV	Flat	YA v. OA	166	0.10	0.920	
	Low	YA v. OA	166	-0.14	0.889	
	Med	YA v. OA	166	-0.56	0.576	
	High	YA v. OA	166	-2.74	0.007	**

Band	Group	Terrain	Z value	pFDR- adjusted	Sig
Step Duration CoV	YA v. OA	Low v. Flat	-0.30	0.765	
	YA v. OA	Med v. Flat	-0.82	0.765	
	YA v. OA	High v. Flat	-3.52	0.003	**
	YA v. OA	Med v. Low	-0.52	0.765	
	YA v. OA	High v. Low	-3.22	0.006	**
	YA v. OA	High v. Med	-2.70	0.027	*

Supplementary Table 3: Multiple comparison results for anteroposterior sacral excursion CoV with significant terrain effect (*p<0.05, **p<0.01, ***p<0.001).

Brain Area	Terrain	dF	t	pFDR- adjusted	Sig
Anteroposterior Excursion CoV	Flat v. Low	300	-7.467	<0.001	***
	Flat v. Med	300	-10.839	<0.001	***
	Flat v. High	300	-15.809	<0.001	***
	Low v. Med	300	-3.371	<0.001	***
	Low v. High	300	-8.342	<0.001	***
	Med v. High	300	-4.971	<0.001	***

Supplementary Table 4: Multiple comparison results for mediolateral sacral excursion CoV with significant interaction effect (*p<0.05, **p<0.01, ***p<0.001).

Measure	Group	Terrain	dF	t	pFDR- adjusted	Sig
Mediolateral Excursion CoV	YA	Flat v. Low	297	-2.21	0.084	
		Flat v. Med	297	-3.57	0.002	**
		Flat v. High	297	-5.43	<0.001	***
		Low v. Med	297	-1.36	0.174	
		Low v. High	297	-3.22	0.006	**
		Med v. High	297	-1.86	0.127	
	OA	Flat v. Low	297	-6.77	<0.001	***
		Flat v. Med	297	-9.50	<0.001	***
		Flat v. High	297	-14.28	<0.001	***
		Low v. Med	297	-2.73	0.007	**
		Low v. High	297	-7.52	<0.001	***
		Med v. High	297	-4.79	<0.001	***

Band	Terrain	Group	dF	t	pFDR- adjusted	Sig
Mediolateral Excursion CoV	Flat	YA v. OA	145	0.55	0.585	
	Low	YA v. OA	145	-0.79	0.428	
	Med	YA v. OA	145	-1.06	0.290	
	High	YA v. OA	145	-1.84	0.068	

Band	Group	Terrain	Z value	pFDR- adjusted	Sig
Mediolateral Excursion CoV	YA v. OA	Low v. Flat	-1.91	0.224	
	YA v. OA	Med v. Flat	-2.29	0.110	
	YA v. OA	High v. Flat	-3.39	0.004	**
	YA v. OA	Med v. Low	-0.38	0.704	
	YA v. OA	High v. Low	-1.48	0.415	
	YA v. OA	High v. Med	-1.10	0.541	

Supplementary Table 5: Full statistical table for power spectral densities at theta, alpha, beta band at each brain area. (SMA: sensorimotor; PP: posterior parietal; MCC: mid-cingulate; Pre-SMA: pre-supplementary motor)

Brain Area	Band	Effect	Fstats	dF	dF(res)	Pvalue	Sig	Partial eta^2
SMA L	Theta	Terrain	0.94	3	225	0.421		0.012
		Age	5.25	1	73	0.025	*	0.067
		Treadmill Speed	0.46	1	73	0.502		0.006
	Alpha	Terrain	18.31	3	225	< 0.001	***	0.196
		Age	0.28	1	73	0.598		0.004
		Treadmill Speed	0.61	1	73	0.438		0.008
	Beta	Terrain	27.42	3	225	< 0.001	***	0.268
		Age	1.15	1	73	0.288		0.015
		Treadmill Speed	1.79	1	73	0.185		0.024
SMA R	Theta	Terrain	0.79	3	189	0.5		0.012
		Age	5.85	1	61	0.019	*	0.087
		Treadmill Speed	1.91	1	61	0.172		0.03
	Alpha	Terrain	18.85	3	189	< 0.001	***	0.23
		Age	1.33	1	61	0.253		0.021
		Treadmill Speed	0.06	1	61	0.8		0.001
	Beta	Terrain	19.14	3	189	< 0.001	***	0.233
		Age	0.37	1	61	0.545		0.006
		Treadmill Speed	0.03	1	61	0.864		0
PP L	Theta	Terrain	5.86	3	207	0.001	***	0.078
		Age	0.24	1	67	0.623		0.004
		Treadmill Speed	0.65	1	67	0.424		0.01
	Alpha	Terrain	29.66	3	204	< 0.001	***	0.304
		Age	0.36	1	67	0.553		0.005
		Treadmill Speed	0.69	1	67	0.409		0.01
		Terrain:Age	9.26	3	204	< 0.001	***	0.12
	Beta	Terrain	37.37	3	207	< 0.001	***	0.351
		Age	0.29	1	67	0.592		0.004
		Treadmill Speed	2.01	1	67	0.161		0.029
PP R	Theta	Terrain	3.97	3	171	0.009	**	0.065
		Age	0.05	1	55	0.829		0.001
		Treadmill Speed	1.56	1	55	0.218		0.028
	Alpha	Terrain	18.32	3	168	< 0.001	***	0.247

		Age	0.22	1	55	0.642		0.004
		Treadmill Speed	0.09	1	55	0.759		0.002
		Terrain:Age	3.63	3	168	0.014	*	0.061
	Beta	Terrain	43.32	3	168	< 0.001	***	0.436
		Age	1.61	1	55	0.209		0.029
		Treadmill Speed	0.06	1	55	0.809		0.001
		Terrain:Age	3.38	3	168	0.02	*	0.057
MCC	Theta	Terrain	3.59	3	177	0.015	*	0.057
		Age	5.38	1	57	0.024	*	0.086
		Treadmill Speed	0.59	1	57	0.445		0.01
	Alpha	Terrain	4.16	3	177	0.007	**	0.066
		Age	0.11	1	57	0.744		0.002
		Treadmill Speed	0.06	1	57	0.805		0.001
	Beta	Terrain	20.81	3	177	< 0.001	***	0.261
		Age	4.71	1	57	0.034	*	0.076
		Treadmill Speed	0.79	1	57	0.379		0.014
Occipital	Theta	Terrain	29.46	3	225	< 0.001	***	0.282
		Age	1.1	1	73	0.298		0.015
		Treadmill Speed	2.19	1	73	0.143		0.029
	Alpha	Terrain	2.84	3	222	0.039	*	0.037
		Age	0.71	1	73	0.402		0.01
		Treadmill Speed	0.12	1	73	0.729		0.002
		Terrain:Age	5.86	3	222	0.001	***	0.073
	Beta	Terrain	47.75	3	222	< 0.001	***	0.392
		Age	0.37	1	73	0.548		0.005
		Treadmill Speed	0.33	1	73	0.569		0.004
		Terrain:Age	6.36	3	222	< 0.001	***	0.079
Pre-SMA	Theta	Terrain	0.27	3	174	0.845		0.005
		Age	0.68	1	56	0.414		0.012
		Treadmill Speed	0	1	56	0.98		0
	Alpha	Terrain	0.88	3	171	0.454		0.015
		Age	0.6	1	56	0.44		0.011
		Treadmill Speed	1.26	1	56	0.267		0.022
		Terrain:Age	2.83	3	171	0.04	*	0.047
	Beta	Terrain	8.64	3	174	< 0.001	***	0.13
		Age	0.33	1	56	0.566		0.006

		Treadmill Speed	0.7	1	56	0.405		0.012
Premotor	Theta	Terrain	1.03	3	177	0.379		0.017
		Age	1.76	1	57	0.19		0.03
		Treadmill Speed	0.07	1	57	0.79		0.001
	Alpha	Terrain	5.7	3	177	0.001	***	0.088
		Age	0.34	1	57	0.563		0.006
		Treadmill Speed	0.01	1	57	0.933		0
	Beta	Terrain	7.86	3	177	< 0.001	***	0.118
		Age	0.03	1	57	0.874		0
		Treadmill Speed	4.54	1	57	0.038	*	0.074

Supplementary Table 6: Multiple comparisons between terrain conditions for each brain cluster that shows a significant terrain effect (*p<0.05, **p<0.01, ***p<0.001).

Band	Brain Area	Terrain	dF	t	pFDR- adjusted	Sig
Theta	PP L	Flat v. Low	207	0.73	0.524	
		Flat v. Med	207	-0.64	0.524	
		Flat v. High	207	-3.21	0.008	**
		Low v. Med	207	-1.37	0.519	
		Low v. High	207	-3.94	0.001	***
		Med v. High	207	-2.57	0.044	*
	PP R	Flat v. Low	171	-0.76	0.450	
		Flat v. Med	171	-1.53	0.384	
		Flat v. High	171	-3.29	0.007	**
		Low v. Med	171	-0.77	0.450	
		Low v. High	171	-2.53	0.061	
		Med v. High	171	-1.76	0.321	
	MCC	Flat v. Low	177	-0.84	0.785	
		Flat v. Med	177	-2.52	0.063	
		Flat v. High	177	-2.79	0.035	*
		Low v. Med	177	-1.68	0.286	
		Low v. High	177	-1.95	0.211	
		Med v. High	177	-0.27	0.785	
	Occipital	Flat v. Low	225	-3.84	<0.001	***
		Flat v. Med	225	-6.92	<0.001	***
		Flat v. High	225	-8.77	<0.001	***
		Low v. Med	225	-3.07	0.005	**
		Low v. High	225	-4.93	<0.001	***
		Med v. High	225	-1.86	0.065	
**Band**	**Brain Area**	**Terrain**	**dF**	**t**	**pFDR- adjusted**	**Sig**
Alpha	SMA L	Flat v. Low	225	4.18	<0.001	***
		Flat v. Med	225	5.17	<0.001	***
		Flat v. High	225	7.18	<0.001	***
		Low v. Med	225	1.00	0.321	
		Low v. High	225	3.01	0.009	**
		Med v. High	225	2.01	0.091	
	SMA R	Flat v. Low	189	3.70	0.001	**
		Flat v. Med	189	5.37	<0.001	***
		Flat v. High	189	7.22	<0.001	***
		Low v. Med	189	1.66	0.098	
		Low v. High	189	3.51	0.002	**
		Med v. High	189	1.85	0.098	

	MCC	Flat v. Low	177	2.52	0.063	
		Flat v. Med	177	1.82	0.285	
		Flat v. High	177	3.40	0.005	**
		Low v. Med	177	-0.70	0.483	
		Low v. High	177	0.88	0.483	
		Med v. High	177	1.58	0.346	
	Premotor	Flat v. Low	177	2.66	0.034	*
		Flat v. Med	177	2.88	0.022	*
		Flat v. High	177	3.98	0.001	***
		Low v. Med	177	0.22	0.829	
		Low v. High	177	1.31	0.548	
		Med v. High	177	1.10	0.548	
**Band**	**Brain Area**	**Terrain**	**dF**	**t**	**pFDR- adjusted**	**Sig**
Beta	SMA L	Flat v. Low	225	4.31	<0.001	***
		Flat v. Med	225	6.38	<0.001	***
		Flat v. High	225	8.72	<0.001	***
		Low v. Med	225	2.07	0.040	*
		Low v. High	225	4.42	<0.001	***
		Med v. High	225	2.35	0.040	*
	SMA R	Flat v. Low	189	3.70	0.001	***
		Flat v. Med	189	5.08	<0.001	***
		Flat v. High	189	7.39	<0.001	***
		Low v. Med	189	1.38	0.169	
		Low v. High	189	3.69	0.001	***
		Med v. High	189	2.31	0.044	*
	PP L	Flat v. Low	207	4.86	<0.001	***
		Flat v. Med	207	8.04	<0.001	***
		Flat v. High	207	9.87	<0.001	***
		Low v. Med	207	3.18	0.003	**
		Low v. High	207	5.01	<0.001	***
		Med v. High	207	1.83	0.069	
	MCC	Flat v. Low	177	3.21	0.003	**
		Flat v. Med	177	3.27	0.003	**
		Flat v. High	177	7.84	<0.001	***
		Low v. Med	177	0.06	0.953	
		Low v. High	177	4.63	<0.001	***
		Med v. High	177	4.57	<0.001	***
	Pre-SMA	Flat v. Low	174	1.91	0.162	
		Flat v. Med	174	3.31	0.006	**
		Flat v. High	174	4.89	<0.001	***

		Low v. Med	174	1.40	0.162	
		Low v. High	174	2.98	0.013	*
		Med v. High	174	1.58	0.162	
	Premotor	Flat v. Low	177	2.33	0.071	
		Flat v. Med	177	3.55	0.002	**
		Flat v. High	177	4.62	<0.001	***
		Low v. Med	177	1.22	0.288	
		Low v. High	177	2.28	0.071	
		Med v. High	177	1.07	0.288	

Supplementary Table 7: Multiple comparison results for left posterior parietal alpha band with significant interaction effect (*p<0.05, **p<0.01, ***p<0.001).

Band	Brain Area	Group	Terrain	dF	t	pFDR- adjusted	Sig
Alpha	PP L	YA	Flat v. Low	204	4.92	<0.001	***
			Flat v. Med	204	8.21	<0.001	***
			Flat v. High	204	7.79	<0.001	***
			Low v. Med	204	3.29	0.004	**
			Low v. High	204	2.87	0.009	**
			Med v. High	204	-0.43	0.671	
		OA	Flat v. Low	204	1.61	0.217	
			Flat v. Med	204	2.18	0.121	
			Flat v. High	204	3.80	0.001	**
			Low v. Med	204	0.57	0.570	
			Low v. High	204	2.19	0.121	
			Med v. High	204	1.62	0.217	

Band	Brain Area	Terrain	Group	dF	t	pFDR- adjusted	Sig
Alpha	PP L	Flat	YA v. OA	76.48	1.79	0.077	
		Low	YA v. OA	76.48	0.60	0.548	
		Med	YA v. OA	76.48	-0.32	0.749	
		High	YA v. OA	76.48	0.23	0.815	

Band	Brain Area	Group	Terrain	Z value	pFDR- adjusted	Sig
Alpha	PP L	YA v. OA	Low v. Flat	-2.85	0.017	*
		YA v. OA	Med v. Flat	-5.08	<0.001	***
		YA v. OA	High v. Flat	-3.74	<0.001	***
		YA v. OA	Med v. Low	-2.23	0.078	
		YA v. OA	High v. Low	-0.89	0.374	
		YA v. OA	High v. Med	1.34	0.362	

Supplementary Table 8: Multiple comparison results for right posterior parietal alpha band with significant interaction effect (*p<0.05, **p<0.01, ***p<0.001).

Band	Brain Area	Group	Terrain	dF	t	pFDR- adjusted	Sig
Alpha	PP R	YA	Flat v. Low	168	4.02	<0.001	***
			Flat v. Med	168	6.15	<0.001	***
			Flat v. High	168	6.13	<0.001	***
			Low v. Med	168	2.13	0.073	
			Low v. High	168	2.11	0.073	
			Med v. High	168	-0.02	0.984	
		OA	Flat v. Low	168	1.81	0.291	
			Flat v. Med	168	2.49	0.068	
			Flat v. High	168	2.95	0.022	*
			Low v. Med	168	0.69	0.648	
			Low v. High	168	1.15	0.648	
			Med v. High	168	0.46	0.648	

Band	Brain Area	Terrain	Group	dF	t	pFDR- adjusted	Sig
Alpha	PP R	Flat	YA v. OA	64.76	1.32	0.193	
		Low	YA v. OA	64.76	0.47	0.643	
		Med	YA v. OA	64.76	-0.07	0.947	
		High	YA v. OA	64.76	0.08	0.938	

Band	Brain Area	Group	Terrain	Z value	pFDR- adjusted	Sig
Alpha	PP R	YA v. OA	Low v. Flat	-1.84	0.260	
		YA v. OA	Med v. Flat	-3.00	0.016	*
		YA v. OA	High v. Flat	-2.68	0.036	*
		YA v. OA	Med v. Low	-1.16	0.743	
		YA v. OA	High v. Low	-0.84	0.752	
		YA v. OA	High v. Med	0.32	0.752	

Supplementary Table 9: Multiple comparison results for occipital alpha band with significant interaction effect (*p<0.05, **p<0.01, ***p<0.001).

Band	Brain Area	Group	Terrain	dF	t	pFDR- adjusted	Sig
Alpha	Occipital	YA	Flat v. Low	222	3.45	0.004	**
			Flat v. Med	222	3.26	0.006	**
			Flat v. High	222	3.20	0.006	**
			Low v. Med	222	-0.19	0.951	
			Low v. High	222	-0.25	0.951	
			Med v. High	222	-0.06	0.951	
		OA	Flat v. Low	222	0.15	0.880	
			Flat v. Med	222	-1.47	0.427	
			Flat v. High	222	-1.93	0.276	
			Low v. Med	222	-1.62	0.424	
			Low v. High	222	-2.08	0.233	
			Med v. High	222	-0.46	0.880	

Band	Brain Area	Terrain	Group	dF	t	pFDR- adjusted	Sig
Alpha	Occipital	Flat	YA v. OA	97.01	0.71	0.478	
		Low	YA v. OA	97.01	-0.92	0.360	
		Med	YA v. OA	97.01	-1.40	0.165	
		High	YA v. OA	97.01	-1.53	0.129	

Band	Brain Area	Group	Terrain	Z value	pFDR- adjusted	Sig
Alpha	Occipital	YA v. OA	Low v. Flat	-2.71	0.027	*
		YA v. OA	Med v. Flat	-3.51	0.002	**
		YA v. OA	High v. Flat	-3.72	0.001	**
		YA v. OA	Med v. Low	-0.80	0.829	
		YA v. OA	High v. Low	-1.01	0.829	
		YA v. OA	High v. Med	-0.22	0.829	

Supplementary Table 10: Multiple comparison results for left pre-supplementary motor band with significant interaction effect (*p<0.05, **p<0.01, ***p<0.001).

Band	Brain Area	Group	Terrain	dF	t	pFDR- adjusted	Sig
Alpha	Pre-SMA	YA	Flat v. Low	171	0.38	0.707	
			Flat v. Med	171	1.21	0.707	
			Flat v. High	171	-0.80	0.707	
			Low v. Med	171	0.83	0.707	
			Low v. High	171	-1.18	0.707	
			Med v. High	171	-2.01	0.279	
		OA	Flat v. Low	171	-0.54	0.587	
			Flat v. Med	171	0.65	0.587	
			Flat v. High	171	2.06	0.203	
			Low v. Med	171	1.20	0.587	
			Low v. High	171	2.61	0.060	
			Med v. High	171	1.41	0.587	

Band	Brain Area	Terrain	Group	dF	t	pFDR- adjusted	Sig
Alpha	Pre-SMA	Flat	YA v. OA	61.18	-0.83	0.411	
		Low	YA v. OA	61.18	-1.04	0.300	
		Med	YA v. OA	61.18	-1.00	0.320	
		High	YA v. OA	61.18	-0.17	0.866	

Band	Brain Area	Group	Terrain	Z value	pFDR- adjusted	Sig
Alpha	Pre-SMA	YA v. OA	Low v. Flat	-0.64	0.901	
		YA v. OA	Med v. Flat	-0.51	0.901	
		YA v. OA	High v. Flat	1.93	0.214	
		YA v. OA	Med v. Low	0.12	0.901	
		YA v. OA	High v. Low	2.57	0.061	
		YA v. OA	High v. Med	2.44	0.073	

Supplementary Table 11: Multiple comparison results for right posterior parietal beta band with significant interaction effect (*p<0.05, **p<0.01, ***p<0.001).

Band	Brain Area	Group	Terrain	dF	t	pFDR- adjusted	Sig
Beta	PP R	YA	Flat v. Low	168	2.83	0.011	*
			Flat v. Med	168	7.17	<0.001	***
			Flat v. High	168	8.11	<0.001	***
			Low v. Med	168	4.34	<0.001	***
			Low v. High	168	5.28	<0.001	***
			Med v. High	168	0.94	0.348	
		OA	Flat v. Low	168	4.27	<0.001	***
			Flat v. Med	168	5.33	<0.001	***
			Flat v. High	168	6.54	<0.001	***
			Low v. Med	168	1.06	0.291	
			Low v. High	168	2.26	0.075	
			Med v. High	168	1.20	0.291	

Band	Brain Area	Terrain	Group	dF	t	pFDR- adjusted	Sig
Beta	PP R	Flat	YA v. OA	61.61	1.53	0.131	
		Low	YA v. OA	61.61	1.79	0.079	
		Med	YA v. OA	61.61	0.79	0.430	
		High	YA v. OA	61.61	0.83	0.412	

Band	Brain Area	Group	Terrain	Z value	pFDR- adjusted	Sig
Beta	PP R	YA v. OA	Low v. Flat	0.67	0.935	
		YA v. OA	Med v. Flat	-1.90	0.205	
		YA v. OA	High v. Flat	-1.82	0.205	
		YA v. OA	Med v. Low	-2.58	0.059	
		YA v. OA	High v. Low	-2.50	0.062	
		YA v. OA	High v. Med	0.08	0.935	

Supplementary Table 12: Multiple comparison results for occipital beta band with significant interaction effect (*p<0.05, **p<0.01, ***p<0.001).

Band	Brain Area	Group	Terrain	dF	t	pFDR- adjusted	Sig
Beta	Occipital	YA	Flat v. Low	222	6.64	<0.001	***
			Flat v. Med	222	8.36	<0.001	***
			Flat v. High	222	8.77	<0.001	***
			Low v. Med	222	1.72	0.174	
			Low v. High	222	2.13	0.102	
			Med v. High	222	0.42	0.678	
		OA	Flat v. Low	222	5.32	<0.001	***
			Flat v. Med	222	4.74	<0.001	***
			Flat v. High	222	6.20	<0.001	***
			Low v. Med	222	-0.59	0.559	
			Low v. High	222	0.88	0.559	
			Med v. High	222	1.46	4.359	

Band	Brain Area	Terrain	Group	dF	t	pFDR- adjusted	Sig
Beta	Occipital	Flat	YA v. OA	87.35	1.75	0.083	
		Low	YA v. OA	87.35	0.66	0.511	
		Med	YA v. OA	87.35	-0.18	0.859	
		High	YA v. OA	87.35	0.07	0.943	

Band	Brain Area	Group	Terrain	Z value	pFDR- adjusted	Sig
Beta	Occipital	YA v. OA	Low v. Flat	-2.27	0.093	
		YA v. OA	Med v. Flat	-4.01	<0.001	***
		YA v. OA	High v. Flat	-3.49	0.002	**
		YA v. OA	Med v. Low	-1.74	0.247	
		YA v. OA	High v. Low	-1.22	0.446	
		YA v. OA	High v. Med	0.52	0.604	

Supplementary Table 13 Full statistical table for power fluctuations at theta, alpha, beta band at each brain area. (SMA: sensorimotor; PP: posterior parietal; MCC: mid-cingulate; Pre-SMA: pre-supplementary motor)

Brain Area	Band	Effect	Fstats	dF	dF(res)	Pvalue	Sig	Partial eta^2
SMA L	theta	Terrain	0.41	3	225	0.745		0.005
		Age	1.29	1	73	0.261		0.017
		Treadmill Speed	1.03	1	73	0.314		0.014
	alpha	Terrain	1.59	3	225	0.192		0.021
		Age	2.79	1	73	0.099		0.037
		Treadmill Speed	0.34	1	73	0.563		0.005
	beta	Terrain	5.78	3	225	0.001	***	0.072
		Age	1.34	1	73	0.251		0.018
		Treadmill Speed	2.22	1	73	0.141		0.029
SMA R	theta	Terrain	0.25	3	188	0.86		0.004
		Age	4.24	1	61	0.044	*	0.065
		Treadmill Speed	0.29	1	61	0.592		0.005
	alpha	Terrain	1.68	3	189	0.173		0.026
		Age	16.2	1	61	< 0.001	***	0.21
		Treadmill Speed	0.15	1	61	0.699		0.002
	beta	Terrain	0.89	3	189	0.449		0.014
		Age	3.47	1	61	0.067		0.054
		Treadmill Speed	0.09	1	61	0.77		0.001
PP L	theta	Terrain	0.31	3	206	0.82		0.004
		Age	0.55	1	67	0.459		0.008
		Treadmill Speed	0.16	1	67	0.694		0.002
	alpha	Terrain	0.84	3	206	0.471		0.012
		Age	3.85	1	67	0.054		0.054
		Treadmill Speed	0.75	1	67	0.388		0.011
	beta	Terrain	0.47	3	206	0.706		0.007
		Age	4.97	1	67	0.029	*	0.069
		Treadmill Speed	4.77	1	67	0.033	*	0.066
PP R	theta	Terrain	0.34	3	171	0.795		0.006
		Age	0.12	1	55	0.735		0.002
		Treadmill Speed	2.52	1	55	0.118		0.044
	alpha	Terrain	0.59	3	171	0.623		0.01
		Age	4.77	1	55	0.033	*	0.08
		Treadmill Speed	5.81	1	55	0.019	*	0.096
	beta	Terrain	0.47	3	171	0.702		0.008
		Age	12.18	1	55	0.001	***	0.181

		Treadmill Speed	2.39	1	55	0.128		0.042
MCC	theta	Terrain	0.06	3	177	0.978		0.001
		Age	0.02	1	57	0.892		0
		Treadmill Speed	0.08	1	57	0.782		0.001
	alpha	Terrain	0.82	3	177	0.487		0.014
		Age	0.65	1	57	0.423		0.011
		Treadmill Speed	0.04	1	57	0.852		0.001
	beta	Terrain	0.95	3	176	0.419		0.016
		Age	0.03	1	57	0.864		0.001
		Treadmill Speed	0.2	1	57	0.656		0.004
Occipital	theta	Terrain	1.99	3	221	0.117		0.026
		Age	1.65	1	73	0.203		0.022
		Treadmill Speed	1.47	1	73	0.229		0.02
		Terrain:Age	4.8	3	221	0.003	**	0.061
	alpha	Terrain	0.82	3	224	0.485		0.011
		Age	3.29	1	73	0.074		0.043
		Treadmill Speed	2.52	1	73	0.117		0.033
	beta	Terrain	1.52	3	221	0.209		0.02
		Age	13.36	1	73	< 0.001	***	0.155
		Treadmill Speed	4.1	1	73	0.047	*	0.053
		Terrain:Age	3.24	3	221	0.023	*	0.042
Pre-SMA	theta	Terrain	0.79	3	174	0.501		0.013
		Age	1.51	1	56	0.224		0.026
		Treadmill Speed	0.12	1	56	0.727		0.002
	alpha	Terrain	0.04	3	174	0.99		0.001
		Age	1.38	1	56	0.245		0.024
		Treadmill Speed	6.39	1	56	0.014	*	0.102
	beta	Terrain	1.49	3	174	0.22		0.025
		Age	2.92	1	56	0.093		0.05
		Treadmill Speed	5.04	1	56	0.029	*	0.083
Premotor	theta	Terrain	0.64	3	177	0.588		0.011
		Age	0.48	1	57	0.489		0.008
		Treadmill Speed	0.41	1	57	0.525		0.007
	alpha	Terrain	0.52	3	177	0.667		0.009
		Age	0.43	1	57	0.514		0.008
		Treadmill Speed	3.06	1	57	0.085		0.051
	beta	Terrain	0.89	3	177	0.448		0.015
		Age	1.8	1	57	0.185		0.031
		Treadmill Speed	2.65	1	57	0.109		0.044

Supplementary Table 14: Multiple comparison results for occipital theta band power fluctuations with significant interaction effect (*p<0.05).

Band	Brain Area	Group	Terrain	dF	t	pFDR- adjusted	Sig
Theta	Occipital	YA	Flat v. Low	221	-2.50	0.053	
			Flat v. Med	221	-3.15	0.009	**
			Flat v. High	221	-3.28	0.007	**
			Low v. Med	221	-0.65	0.891	
			Low v. High	221	-0.79	0.891	
			Med v. High	221	-0.14	0.891	
		OA	Flat v. Low	221	0.84	0.713	
			Flat v. Med	221	1.64	0.618	
			Flat v. High	221	0.48	0.713	
			Low v. Med	221	0.80	0.713	
			Low v. High	221	-0.37	0.713	
			Med v. High	221	-1.17	0.713	

Band	Brain Area	Terrain	Group	dF	t	pFDR- adjusted	Sig
Theta	Occipital	Flat	YA v. OA	149	-1.03	0.303	
		Low	YA v. OA	149	1.31	0.192	
		Med	YA v. OA	149	2.24	0.027	*
		High	YA v. OA	149	1.71	0.090	

Band	Brain Area	Group	Terrain	Z value	pFDR- adjusted	Sig
Theta	Occipital	YA v. OA	Low v. Flat	2.52	0.047	*
		YA v. OA	Med v. Flat	3.52	0.003	**
		YA v. OA	High v. Flat	2.94	0.016	*
		YA v. OA	Med v. Low	1.00	0.672	
		YA v. OA	High v. Low	0.42	0.672	
		YA v. OA	High v. Med	-0.58	0.672	

Supplementary Table 15: Multiple comparison results for occipital beta band power fluctuations with significant interaction effect (*p<0.05, **p<0.01, ***p<0.001).

Band	Brain Area	Group	Terrain	dF	t	pFDR- adjusted	Sig
Beta	Occipital	YA	Low v. Flat	221	-1.61	0.437	
			Med v. Flat	221	-2.32	0.106	
			High v. Flat	221	-2.86	0.028	*
			Med v. Low	221	-0.71	0.590	
			High v. Low	221	-1.25	0.590	
			High v. Med	221	-0.54	0.590	
		OA	Low v. Flat	221	1.69	0.556	
			Med v. Flat	221	0.50	0.619	
			High v. Flat	221	1.03	0.619	
			Med v. Low	221	-1.20	0.619	
			High v. Low	221	-0.66	0.619	
			High v. Med	221	0.54	0.619	

Band	Brain Area	Terrain	Group	dF	t	pFDR- adjusted	Sig
Beta	Occipital	Flat	YA v. OA	155	1.20	0.234	
		Low	YA v. OA	155	3.39	0.001	***
		Med	YA v. OA	155	3.28	0.001	**
		High	YA v. OA	155	3.99	< 0.001	***

Band	Brain Area	Group	Terrain	Z value	pFDR- adjusted	Sig
Beta	Occipital	YA v. OA	Low v. Flat	2.30	0.109	
		YA v. OA	Med v. Flat	2.17	0.119	
		YA v. OA	High v. Flat	2.92	0.021	*
		YA v. OA	Med v. Low	-0.12	0.902	
		YA v. OA	High v. Low	0.63	0.902	
		YA v. OA	High v. Med	0.75	0.902	
